# Emotional Contagion is not Altered in Mice Prenatally Exposed to Poly (I:C) on Gestational Day 9

**DOI:** 10.3389/fnbeh.2016.00134

**Published:** 2016-06-28

**Authors:** Cristina Gonzalez-Liencres, Georg Juckel, Manuela Esslinger, Simone Wachholz, Marie-Pierre Manitz, Martin Brüne, Astrid Friebe

**Affiliations:** ^1^Department of Psychiatry, LWL University Hospital, Ruhr-University BochumBochum, Germany; ^2^International Graduate School of Neuroscience, Ruhr-University BochumBochum, Germany

**Keywords:** schizophrenia, empathy, maternal immune activation, Poly(I:C), social behavior

## Abstract

Prenatal immune activation has been associated with increased risk of developing schizophrenia. The polyinosinic-polycytidylic acid (Poly(I:C)) mouse model replicates some of the endophenotype characteristic of this disorder but the social deficits observed in schizophrenia patients have not been well studied in this model. Therefore we aimed to investigate social behavior, in particular emotional contagion for pain, in this mouse model. We injected pregnant mouse dams with Poly(I:C) or saline (control) on gestation day 9 (GD9) and we evaluated their offspring in the pre-pulse inhibition (PPI) test at age 50–55 days old to confirm the reliability of our model. Mice were then evaluated in an emotional contagion test immediately followed by the light/dark test to explore post-test anxiety-like behavior at 10 weeks of age. In the emotional contagion test, an observer (prenatally exposed to Poly(I:C) or to saline) witnessed a familiar wild-type (WT) mouse (demonstrator) receiving electric foot shocks. Our results replicate the sensory gating impairments in the Poly(I:C) offspring but we only observed minor group differences in the social tasks. One of the differences we found was that demonstrators deposited fewer feces in the presence of control observers than of observers prenatally exposed to Poly(I:C), which we suggest could be due to the observers’ behavior. We discuss the findings in the context of age, sex and day of prenatal injection, suggesting that Poly(I:C) on GD9 may be a valuable tool to assess other symptoms or symptom clusters of schizophrenia but perhaps not comprising the social domain.

## Introduction

Prenatal immunological processes interfere with normal neurodevelopment thereby increasing the risk of developing severe mental illness later in life, such as schizophrenia (Fatemi et al., 2008). In particular, prenatal infection in rodents leads to an overactivation of the immune system characterized by increased activated microglia (brain immune cells; Li et al., 2014; Zhu et al., 2014). In humans, PET scans and post-mortem studies have shown increased microglia in schizophrenia patients (Bayer et al., 1999; Radewicz et al., 2000). Thus, findings in mice and humans point to a potential active role of microglia and immunological processes in the pathogenesis of neuropsychiatric disorders (Juckel et al., 2011; Manitz et al., 2013; Juckel, 2015).

Animal models that mimic some of the symptoms or symptom clusters of schizophrenia have been developed by manipulating immunodevelopmental factors, such as the polyinosinic-polycytidylic acid (Poly(I:C)) model, which is produced by injecting Poly(I:C) in a pregnant mouse dam to generate an immunological response that will affect the neurodevelopment of the pups.

Prenatal exposure to Poly (I:C) results in different phenotypes depending on the time of the exposure. When Poly(I:C) is injected on gestation day 9 (GD9; corresponding roughly to middle-to-late first trimester of pregnancy in humans; Clancy et al., 2007), the pups develop dysfunctions in sensorimotor gating, latent inhibition, exploratory behavior and working memory, increased sensitivity to amphetamine, and a general hyperdopaminergic state in mesocorticolimbic regions (Meyer et al., 2005, 2008b; Winter et al., 2009). When Poly(I:C) is injected on GD17, the pups develop other phenotypes, such as social deficits (Bitanihirwe et al., 2010a). Based on these differences between prenatal exposure to Poly(I:C) at GD9 and at GD17, some authors have suggested that these two mouse models could represent two separate symptom clusters of schizophrenia: Poly(I:C) exposure on GD9 could reproduce mostly positive symptoms, while Poly(I:C) exposure on GD17 could reproduce mostly negative symptoms (Sullivan et al., 2006; Meyer and Feldon, 2012). Abnormalities in the neurotransmitter systems in the brain similar to those found in patients with schizophrenia have also been reported in mice prenatally treated with Poly(I:C) (Meyer et al., 2008a,b; Vuillermot et al., 2010).

Despite the fact that social deficits are a feature of schizophrenia, these have not been well studied in the Poly(I:C) model, particularly when injected on GD9. More specifically, to our knowledge empathy and emotional contagion, which are impaired in schizophrenia, have not been studied in any schizophrenia animal model. Previous studies in wild-type (WT) mice, on the other hand, have shown that mice are affected by their cagemate’s stress (Langford et al., 2006; Jeon et al., 2010; Gonzalez-Liencres et al., 2014). For instance, writhing behavior is increased in mice injected with acetic acid when they simultaneously observe another mouse also treated with acetic acid in comparison to when the injected mouse is placed alone or with a non-writhing mouse (Langford et al., 2006). In another task where an observer mouse views another mouse (demonstrator) receiving electric foot shocks in an adjacent compartment, the observer mouse freezes (i.e., a fear response) when the demonstrator receives the shocks (Jeon et al., 2010). Interestingly, the fear or stress response in these emotional contagion tasks in mice increases with familiarity between demonstrator and observer (Langford et al., 2006; Jeon et al., 2010; Gonzalez-Liencres et al., 2014). It has been suggested that the freezing behavior in the observer is induced by a combination of visual, auditory and olfactory (pheromones) cues (Jeon et al., 2010).

The aim of this study was thus to investigate whether prenatal exposure to Poly(I:C) on GD9 would disrupt social abilities in mice, in particular emotional contagion for pain. We hypothesized that the offspring of Poly(I:C)-treated mice would show behavioral differences in sensory gating (reproducing previous findings), in emotional contagion to pain and in post-test anxiety compared with offspring of saline-treated mice. It would be of notable relevance for the scientific community to have an experimental animal model that replicates some of the specific social deficits in patients so that the brain mechanisms and pharmacological alternatives can be explored.

## Materials and Methods

### Mouse Husbandry

C57BL/6J mice were housed in an inverse 12:12 light-dark cycle under pathogen-free, stable temperature (21 ± 1°C) and humidity (50–60%) conditions and were given chow and water *ad libitum*. Half of the mice were used as demonstrators and the other half as observers. Experiments were carried out during the dark (active) phase. Demonstrators and observers were always cagemates and had been living together in same-sex cages for at least 6 weeks prior to the emotional contagion test. All animal procedures were performed in agreement with the European Communities Council Directive of 24 November 1986 (86/609/EEC) and were conducted according to institutional guidelines and after approval by the state authority for animal research conduct of North Rhine–Westphalia, Germany (approval 84-02.04.2014.A135).

### Poly(I:C) vs. Saline Treatment

Demonstrators were WT mice. Observers were offspring of treated mothers, who were given a single i.p. injection of 20 mg/kg Poly(I:C) diluted in saline at a concentration of 2.5 mL/mg on GD9 (Poly(I:C) offspring) or an equivalent i.p. injection of 0.9% saline on GD9 (saline offspring, i.e., controls).

### Sensory Gating

The Pre-Pulse Inhibition (PPI) test was employed to examine sensory gating deficits in mice in a Startle Response box (TSE Systems GmbH, Bad Homburg, Germany). Only observers (7 male and 12 female Poly(I:C) offspring; 9 male and 15 female saline offspring) completed this test at age 50–55 days old. After the mouse was placed in a small restrainer that detects the startle response, there was a baseline period (5 min) with a baseline background noise of 65 dB. The background noise continued throughout the experiment. This was followed by six startle trials to habituate the mice, whereby 120 dB sounds were delivered for 40 ms every 10–20 s. These six startle trials were not used for the analysis. There were then 80 trials, out of which 30 were only pre-pulses for 20 ms (70, 77 and 83 dB); 10 trials were a strong startle (S; 120 dB) for 40 ms; 30 trials were pre-pulse plus startle (PPS), whereby the pre-pulse (70, 77 or 83 dB) lasted 20 ms and after 80 ms the 120 dB startle pulse was delivered for 40 ms; and 10 trials with no stimulus (i.e., background noise, 65 dB) for 100 ms. There was a 1 min recovery period at the end of the experiment. All trials were randomized. The maximal response over the 65 ms after startle stimulus onset was used. PPI percentage %PPI was calculated for each different pre-pulse according to the following formula: % PPI = 100*$\frac{S-PPS}{S}$. Lower %PPI indicates deficits in sensory gating. Global %PPI was the average of the %PPI with the three distinct pre-pulses.

### Emotional Contagion for Pain

Emotional contagion was tested when observers (6 male and 11 female Poly(I:C) offspring; 7 male and 11 female saline offspring) were 10 weeks of age. Each mouse was only used once. A demonstrator WT and an observer (Poly(I:C) offspring or saline offspring) mouse, which were same-sex cagemates, were placed each in one of two chambers of an Active/Passive Avoidance System box (TSE Systems GmbH, Bad Homburg, Germany). The test consisted in a baseline period (5 min) with no shocks, a test period (4 min) in which electric foot shocks (1 s, 0.5 mA) were applied every 10 s through the floor rods exclusively to the demonstrator, and a recovery period (1 min) with no shocks. The test had a total duration of 10 min and observers were filmed with a camera. The apparatus was thoroughly cleaned with 70% ethanol between trials. The parameters measured were the freezing time as an indication of fear (i.e., lack of movement, except for respiration, for longer than 2 s) and the number of fecal droppings (higher number indicatory of fear).

### Post-Test Anxiety

Immediately following the emotional contagion test, observers were placed in a light/dark box (TSE Systems GmbH, Bad Homburg, Germany) to assess the levels of anxiety after witnessing a conspecific receiving foot shocks. The mouse was placed in the dark compartment and was allowed to explore both compartments for 10 min. More time spent in the dark corresponds to increased anxiety-like behavior. The parameters recorded were the amount of time spent in each chamber, the number of entries into each chamber, and the latency to go for the first time from the dark into the light side.

### Statistics

For the statistical analyses, we carried out 3 × 2 × 2 repeated measures analyses of variance (ANOVA) with pre-pulse Intensity (70, 77 and 83 dB) as the within-subjects variable, and Group (Poly(I:C) offspring vs. saline offspring) and Sex (male vs. female) as the between-subjects factors. For freezing time in the emotional contagion test, we performed a 3 × 2 × 2 ANOVA with Period (baseline, test, recovery) as the within-subjects factor, and Group and Sex as the between-subjects variables. Number of fecal droppings in the emotional contagion test, time spent in the dark, number of entries into the dark compartment and latency to go into the light compartment for the first time in the light-dark test were all subjected to two-way ANOVAs with Group and Sex as between-subjects variables. *Post hoc* tests were corrected for multiple comparisons with Bonferroni correction. Spearman correlation analyses were conducted to assess the relationship among variables.

## Results

**Figure 1:**
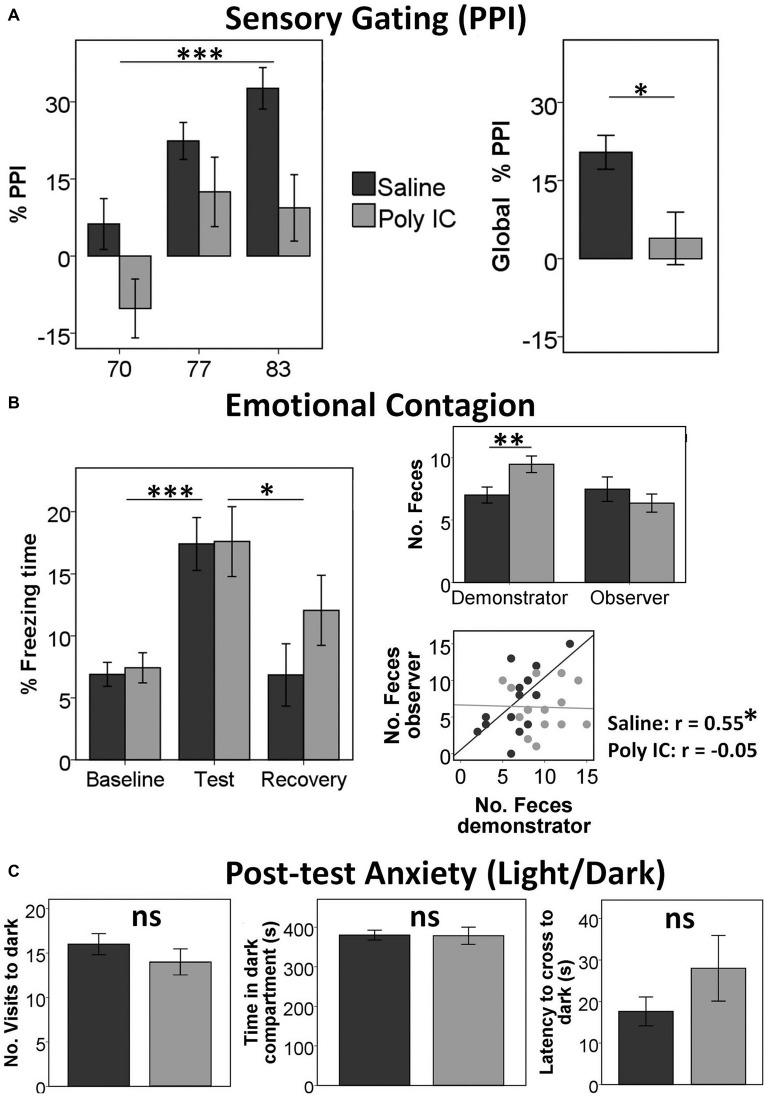
**Offspring of polyinosinic-polycytidylic acid (Poly(I:C))-treated mothers show reduced sensory gating and almost intact social behavior. (A)** Increased %pre-pulse inhibition (PPI) with increased pre-pulse intensity in both groups (left) and reduced Global %PPI in Poly(I:C) offspring (right). **(B)** Freezing (fear-like) behavior in both groups is increased in the test period when the demonstrator is receiving shocks compared with the baseline and the recovery periods (left); wild-type (WT) demonstrators deposit more feces when observer is Poly(I:C) offspring (right). **(C)** No differences in any parameter in the light/dark test measuring anxiety-like behavior in observers after the emotional contagion test. Graphs illustrate Mean ± SEM. *p < 0.05, **p < 0.01, ***p < 0.001.

We first examined whether maternal immune activation with Poly(I:C) induced the expected sensory gating deficits previously reported in other studies. We obtained a significant main effect of Intensity (*F*_(2,38)_ = 18.5, *p* < 0.001, *η*^2^ = 0.49) and a significant main effect of Group (*F*_(1,39)_ = 6.38, *p* = 0.016, *η*^2^ = 0.141), indicating that Poly(I:C) offspring had reduced PPI compared with saline offspring (Mean ± SD: Poly(I:C) offspring *M* = 3.89 ± 22.0; Saline offspring *M* = 20.4 ± 15.9; Figure 1A). We did not find any other significant effects or interactions.

In the emotional contagion test, analysis of the freezing time revealed only a significant main effect of Period (*F*_(2,30)_ = 17.7, *p* < 0.001, *η*^2^ = 0.542; Figure 1B). *Post hoc* tests (corrected *p* = 0.017) showed a significant difference between baseline and test (baseline *M* = 7.15 ± 4.51; test *M* = 17.5 ± 10.2; *t*_(34)_ = −6.19, *p* < 0.001) and between test and recovery (recovery *M* = 9.38 ± 11.3; *t*_(34)_ = 3.782, *p* = 0.001), but not between baseline and recovery (*t*_(34)_ = 1.26, *p* = 0.22). We did not find any other significant main effect or interaction.

In addition, we found that demonstrators WT deposited more feces when the observer was Poly(I:C) offspring (*M* = 9.47 ± 2.74) than when the observer was saline offspring (*M* = 6.72 ± 2.82; *F*_(3,31)_ = 8.44, *p* = 0.007, *η*^2^ = 0.214), but we obtained no differences in the number of feces of the observers (*p* > 0.2). Finally, we observed a correlation between the number of feces of the demonstrators and the observers only when the observers were saline offspring (*r* = 0.55, *p* = 0.018) but not Poly(I:C) offspring (*r* = −0.05, *p* = 0.85; Figure 1C).

Post-test anxiety assessed in the light dark test did not reveal any significant differences between the Poly(I:C) model and the saline controls.

## Discussion

In this study we investigated social behavior, more specifically emotional contagion for pain, in a mouse model of psychiatric disorders usually associated with schizophrenia due to some of the common endophenotypic characteristics with this illness such as alterations in neuroimmunological processes (Juckel et al., 2011; Manitz et al., 2013). Our findings indicate a reduction of PPI in the offspring of Poly(I:C)-treated mothers compared with offspring of saline-treated mothers, small differences in an emotional contagion task, and no effect on anxiety-like behavior after witnessing a conspecific in distress in the emotional contagion test. This study suggests that mice prenatally exposed to Poly(I:C) on GD9 may develop some of the features of schizophrenia but perhaps not in the social domain.

The neurodevelopmental hypothesis of schizophrenia attributes the origin of schizophrenia to developmental insults occurring as early as during pregnancy or birth, such as prenatal exposure to inflammation, with disease onset occurring in late adolescence or early adulthood (Brown, 2006; Fatemi et al., 2008; Fatemi and Folsom, 2009). Accordingly, we employed a mouse model of maternal immune activation, whereby we injected pregnant dams with Poly(I:C), a double-stranded RNA that mimics the immune activation of viruses (Kimura et al., 1994). Poly (I:C) causes an immunological reaction in the injected animal that induces the secretion of cytokines and other molecules. Cytokines have a dual function: they are involved in the immunological reaction but they also contribute to the development of the nervous system. Therefore, when cytokines cross the fetal-maternal barrier, they act on the neurodevelopment of the pups, inducing a series of neurobiological and behavioral abnormalities that resemble those of patients with schizophrenia (Meyer et al., 2006b; Ozawa et al., 2006; Smith et al., 2007; Vuillermot et al., 2010).

In agreement with previous reports (Ozawa et al., 2006; Smith et al., 2007), we found that prenatal exposure to Poly(I:C) reduced PPI, implying deficits in sensory gating in this mouse model. This is paralleled by human epidemiological studies describing impairments in sensory gating in patients with schizophrenia compared with healthy people (Swerdlow et al., 2006). These findings proved that our model was performing as predicted.

Compatible with a previous study by our group (Gonzalez-Liencres et al., 2014), observer mice in the emotional contagion test behaved in the expected pattern: observers expressed low levels of fear (freezing) behavior during the baseline period, which increased during the test period when the mice observed a familiar cagemate demonstrator receiving shocks, and returned to baseline levels after the shocks had ceased in the recovery period. Other studies have found similar behavior in WT mice, whereby freezing behavior increased when the demonstrator was receiving shocks compared with baseline levels, and this effect increased with familiarity (Jeon et al., 2010). This test assesses what some authors call *emotional contagion*, which could be described as a simpler type of empathy that is also present in humans but does not require perspective-taking or high cognitive abilities (Preston and de Waal, 2002; Gonzalez-Liencres et al., 2013). Unexpectedly, we did not find an effect of maternal immune activation on the freezing behavior of the observers when they witnessed a WT conspecific in distress compared to saline-treated control mice. This is in contrast with our hypothesis that offspring of Poly(I:C)-treated mothers would exhibit deficits in this task by showing decreased levels in freezing. Since the “transfer” of distress from the demonstrator to the observer depends on several perceptual cues (visual, auditory and olfactory), it is possible that reducing the communication between the demonstrator and the observer by blocking one of these perceptual cues would have a greater effect on the Poly (I:C) mice than in the saline control mice.

We did find, however, a strong association of the number of fecal droppings between observers and demonstrators in the saline-treated offspring, but not in the Poly(I:C)-treated offspring, which is supported by our previous experiment paralleling these findings in WT familiar pairs of mice (Gonzalez-Liencres et al., 2014). This could denote that mice exposed to prenatal immune activation may show fear behavior to other factors, not necessarily arising from their conspecifics’ distress. In the control group, the association of fear-related parameters (i.e., number of feces) between observer and demonstrator suggest that the degree to which the observers experienced distress was related to that of the demonstrators. These findings are similar to what occurred in the cagemate, but not in the stranger condition in our previous study exploring the influence of familiarity on emotional contagion for pain in mice: observers could be detecting the distress from the demonstrator through different perceptual cues (Gonzalez-Liencres et al., 2014). Moreover, WT demonstrators in the presence of an observer deposited more feces if the observer was Poly(I:C) offspring, perhaps indicating that saline-exposed observers have a *calming* effect on the demonstrators. A potential explanation is that saline-exposed observers act normally and display certain social cues that the demonstrators perceive as coming from a familiar conspecific, as opposed to Poly (I:C)-exposed observers, who may act slightly different and display other types of social cues. Something similar has been reported in humans, whereby the mere presence of a familiar person is able to decrease the distress of another individual (Kissel, 1965). In addition to the emotional contagion test itself, we aimed to assess the levels of anxiety in the observers immediately after being exposed to a conspecific in distress. Contrary to our expectations, we did not find any differences in anxiety-like behavior between mice prenatally exposed to Poly(I:C) and to saline.

Some researchers have recently suggested that Poly(I:C) exposure at distinct prenatal periods may result in different phenotypes. It is plausible that an immune reaction on GD 9 affects neurodevelopmental mechanisms differently from an immune response at another time point, such as GD 17 (Meyer et al., 2006a). More specifically, Poly(I:C) exposure on GD 9 results in dysfunctions in sensorimotor gating, latent inhibition, exploratory behavior and working memory, increased sensitivity to amphetamine, and a general hyperdopaminergic state in mesocorticolimbic regions (Meyer et al., 2005, 2008b; Winter et al., 2009), whereas mice exposed to Poly(I:C) on GD 17 develop social interaction impairments, anhedonic behavior and cognitive deficits, as well as hypodopaminergic and hypoglutamatergic hippocampus and prefrontal cortex (Bitanihirwe et al., 2010a,b). Rooted in this, some authors have proposed that Poly(I:C) exposure on GD 9 may be related to positive symptomatology, while Poly(I:C) exposure on GD 17 may correspond to negative symptoms observed in people with schizophrenia (Sullivan et al., 2006; Meyer and Feldon, 2012). In spite of the challenge to prove this theory, our results support this model since we observed sensorimotor deficits but only minor changes in emotional contagion skills (only apparent as lack of correlation of feces between observers and demonstrators) in mice prenatally exposed to Poly(I:C) on GD 9. For this reason, future research may bring new insights into the mechanisms underlying psychiatric disorders due to maternal immune activation by assessing emotional contagion on mice prenatally exposed to Poly(I:C) on GD17. It should be noted, however, that other explanations are possible. It could be the case that the emotional contagion test that we used in this experiment is not adequate for the study of social deficits in mouse models of psychiatric disorders, and that other tests that assess more general social abilities could detect social deficits that the test we employed could not detect.

This study presents a few limitations. First, the sample size is fairly small and includes both male and female observers. Although we carried out statistical tests taking sex (male vs. female) into account and we did not find any effect, it is worth noting that these results should be sensibly interpreted. Second, mice were 10 weeks of age, i.e., early adulthood, when they were tested for emotional contagion, which may be influenced by age and thus show differences at another age stage. Finally, given the phenotypic differences dependent on the gestational time point when Poly(I:C) is injected, it is possible that Poly(I:C) treatment at another stage (e.g., GD 17) may result in more social-specific behavioral abnormalities. Future research is warranted to explore other variables including discrete age groups, time of Poly(I:C) injection, and the use of opposite sex mice as demonstrators. Finally, research may also benefit from exploring other more basic aspects of social behavior.

In conclusion, our study is the first to provide preliminary experimental evidence for a possible minor role of prenatal immune exposure during early to mid pregnancy on emotional contagion and perhaps other social attributes. Given the slim differences observed in this study, however, the Poly (I:C) on GD 9 mouse model of schizophrenia may not be the most appropriate tool to investigate social behavior. It is worth noting that it is also possible that another type of test that assesses social abilities in mice may be able to detect social deficits that the emotional contagion test that we employed could not detect.

## Author Contributions

CG-L, GJ, MB and AF: designed the study. ME, SW and M-PM: contributed to the implementation of the Poly (I:C) model and the equipment setup. CG-L: collected data, conducted statistical analyses and wrote the first draft of the manuscript. GJ, MB and AF: contributed to the interpretation of the results and the writing of the subsequent drafts of the manuscript. All authors have approved the final manuscript.

## Conflict of Interest Statement

The authors declare that the research was conducted in the absence of any commercial or financial relationships that could be construed as a potential conflict of interest.
